# Identification and Characterization of Breakpoints and Mutations on *Drosophila melanogaster* Balancer Chromosomes

**DOI:** 10.1534/g3.120.401559

**Published:** 2020-09-24

**Authors:** Danny E. Miller, Lily Kahsai, Kasun Buddika, Michael J. Dixon, Bernard Y. Kim, Brian R. Calvi, Nicholas S. Sokol, R. Scott Hawley, Kevin R. Cook

**Affiliations:** * Department of Pediatrics, Division of Genetic Medicine, University of Washington, and Seattle Children’s Hospital, Seattle, WA 98105; † Department of Biology, Indiana University, Bloomington, IN 47405; ‡ Department of Biology, Stanford University, Stanford, CA 94305; § Department of Molecular and Integrative Physiology, University of Kansas Medical Center, Kansas City, KS 66160; ** Stowers Institute for Medical Research, Kansas City, MO 64110

**Keywords:** balancer chromosomes, chromosomal inversions, *p53*, *Ankyrin 2*, *Fucosyltransferase A*

## Abstract

Balancers are rearranged chromosomes used in *Drosophila melanogaster* to maintain deleterious mutations in stable populations, preserve sets of linked genetic elements and construct complex experimental stocks. Here, we assess the phenotypes associated with breakpoint-induced mutations on commonly used third chromosome balancers and show remarkably few deleterious effects. We demonstrate that a breakpoint in *p53* causes loss of radiation-induced apoptosis and a breakpoint in *Fucosyltransferase A* causes loss of fucosylation in nervous and intestinal tissue—the latter study providing new markers for intestinal cell identity and challenging previous conclusions about the regulation of fucosylation. We also describe thousands of potentially harmful mutations shared among *X* or third chromosome balancers, or unique to specific balancers, including an *Ankyrin 2* mutation present on most *TM3* balancers, and reiterate the risks of using balancers as experimental controls. We used long-read sequencing to confirm or refine the positions of two inversions with breakpoints lying in repetitive sequences and provide evidence that one of the inversions, *In(2L)Cy*, arose by ectopic recombination between *foldback* transposon insertions and the other, *In(3R)C*, cleanly separates subtelomeric and telomeric sequences and moves the subtelomeric sequences to an internal chromosome position. In addition, our characterization of *In(3R)C* shows that balancers may be polymorphic for terminal deletions. Finally, we present evidence that extremely distal mutations on balancers can add to the stability of stocks whose purpose is to maintain homologous chromosomes carrying mutations in distal genes. Overall, these studies add to our understanding of the structure, diversity and effectiveness of balancer chromosomes.

Balancer chromosomes occupy an important place in the *Drosophila melanogaster* genetic toolkit. Their extensive rearrangements function both to inhibit meiotic recombination and, when recombination does occur, prevent the recovery of recombinant chromosomes. Usually, the rearrangements that make up the balancer have two or three breakpoints that result in the inversion of chromosomal segments. The frequency of crossing over between a balancer and a normal-sequence homolog is low when the balancer breakpoints are closely linked, but, when the breakpoints are more distantly spaced, two-strand double crossovers can occur and exchange stretches of DNA. Likewise, single crossovers can occur distal to the distalmost breakpoints on chromosome arms if the breakpoints are not close enough to the telomeres. In addition, most balancers carry at least one recessive lethal or sterile mutation to prevent them from outcompeting homologous chromosomes in stock populations, and at least one dominant visible mutation with an easily scored phenotype so they may be tracked in crosses (Miller *et al.* 2019).

Balancers are used most often to maintain stable stocks carrying recessive lethal or sterile mutations and to assure that sets of alleles on homologous chromosomes remain linked together. They are also used in nearly all crosses that generate complex combinations of chromosomes. Because of their incredible usefulness, balancers have been tremendously important in the development of *Drosophila melanogaster* as a genetic model organism.

Despite the widespread use of balancers, the genomic positions of many of the breakpoints of the most commonly used balancers were identified only recently, with many of the breakpoints found to lie within protein-coding genes (Miller *et al.* 2016b, 2016a, 2018a; Ghavi-Helm *et al.* 2019). Miller *et al.* (2018a) assessed the phenotypic consequences of the breakpoints on second chromosome balancers by complementation testing the balancers against chromosomal deletions for breakpoint regions and showed that most breakpoints were not associated with severely deleterious phenotypes, but that the disruption of some genes by breakpoints caused recessive lethality or sterility. For example, the 22E breakpoint on the second chromosome balancer *SM5* disrupts *dachsous*, resulting in lethality with escapers having shortened appendages, and the 22A breakpoint on *SM1*, *SM5* and *SM6a* disrupts *no individualized sperm* resulting in male sterility.

In addition to identifying inversion breakpoints, sequencing the second chromosome balancers *CyO*, *SM5* and *SM6a* identified potentially damaging missense, splice site and nonsense polymorphisms (Miller *et al.* 2018a). Some polymorphisms were shared by some, but not all of the balancers sequenced. For example, all *SM5* and *SM6a* balancers sequenced have a splice-site mutation in *asteroid*, a gene involved in photoreceptor and wing development, that was not observed on any *CyO* balancers. Other polymorphisms appeared to be unique to the balancer from a single stock. For example, the *SM6a* balancer from one particular stock carries a nonsense mutation in the *pickpocket 11* sodium channel gene.

Information on breakpoints and background polymorphisms on balancers is important because it can guide investigators in the choice of balancers for maintaining stocks with mutations in breakpoint-associated genes. It can also alert researchers to potential dose-dependent effects of heterozygous balancer-borne mutations and potential dominant interactions between mutations on balancers and other chromosomes. Moreover, breakpoint information has revealed previously unknown duplicated chromosomal segments such as the region containing 117 protein-coding genes present in two copies on all *SM5* balancers (Miller *et al.* 2018a).

In this study, we use information from sequencing the third chromosome balancers *TM3*, *TM6* and *TM6B* (Miller *et al.* 2016a) to assess the phenotypic effects of breakpoint-associated gene disruptions. We present a survey of the breakpoints on these balancers for severe deleterious phenotypes and demonstrate that breakpoints in the *p53* and *FucTA* genes are associated with more subtle effects that may, nevertheless, be important in many experimental situations. Similar to the previous analysis of second chromosome balancers (Miller *et al.* 2018a), we identify sequence polymorphisms on *X* and third chromosome balancers that may affect protein structure or gene expression. We also provide a characterization of one specific background mutation in the *Ankyrin 2* gene present on many *TM3* balancers.

Previous characterizations of second and third chromosome balancers were unable to localize the breakpoints of two component inversions at single-nucleotide resolution because the breakpoints were associated with repetitive sequences (Miller *et al.* 2016a, 2018a). Here, we refine the genomic positions of the recently mapped *In(3R)C* inversion breakpoints (Ghavi-Helm *et al.* 2019) using long-read, single-molecule sequencing and show that the data provide evidence that balancers may carry terminal deletions. We similarly confirm the genomic positions of the *In(2L)Cy* breakpoints determined by the same investigators and show the inversion arose by ectopic recombination between *foldback* transposons. Finally, we use the genetic characterization of one particular *CyO* balancer to show that deleterious mutations at extremely distal positions can explain the unexpected stability of many stocks.

## Materials and Methods

### Complementation analyses

Fly crosses were made on standard medium, reared under routine conditions and evaluated by customary standards (details provided upon request). In general, complementation results were based on samples exceeding 50 progeny and sterility was judged in tests of >10 progeny. Genomic coordinates are given in terms of the Release 6 assembly. Table S1 provides a list of stocks used and our sources.

### Apoptosis assay

As described previously (Qi and Calvi 2016), young adult females were conditioned on wet yeast for three days and then exposed to 40 Gy of gamma irradiation from a cesium source. Four hours after irradiation, ovaries were dissected and stained by the TUNEL method (*In Situ* Cell Death Detection Kit, TMR red, version 11 (12 156 792 910; Roche, Basel, Switzerland)) and DAPI. Percent of apoptotic cells was determined by scoring TUNEL staining of cells with DAPI-stained nuclei in flattened follicle surfaces, excluding deformed cells at follicle edges and the polar follicle cells that have been shown previously to undergo *p53*-independent, developmentally programmed cell death (Besse and Pret 2003; Mehrotra *et al.* 2008).

### Antibody staining

Brains from adults aged 4–6 days were immunostained as described in Kahsai *et al.* (2010). Briefly, brains were dissected in sodium phosphate-buffered saline with 0.1% Triton X-100 (PBST), fixed in ice-cold 4% paraformaldehyde (Electron Microscopy Services) in sodium phosphate buffer for two hours, rinsed in PBST and incubated with affinity-purified Alexa Fluor 488-conjugated rabbit anti-Horseradish Peroxidase antibody (1:200 dilution) and the mouse nc82 monoclonal antibody (1:10) for 72 hr at 4°. An overnight secondary antibody incubation at 4° with Alexa Fluor 568-conjugated goat anti-mouse antibody (1:1000) was followed by a PBST rinse, a final wash in PBS and mounting in 2.5% (w/v) DABCO (1,4-Diazabicyclo[2.2.2]-octane, Aldrich). The nc82 antibody detects the protein Bruchpilot and provided a counterstain to define overall brain structure by highlighting presynaptic terminals (Rein *et al.* 1999; Wagh *et al.* 2006). Images were collected on a Leica SP5 scanning confocal microscope at the Indiana University Light Microscopy Imaging Center. At least 15 brains were analyzed for each experiment. Images that required comparisons were acquired using the same settings and processed simultaneously using Adobe Photoshop CC.

Adult intestines were immunostained as described in Buddika *et al.* (2020). Briefly, 5–7 day old females were dissected in ice-cold phosphate-buffered saline, and intestines were fixed with ice-cold 4% paraformaldehyde (Electron Microscopy Services) in sodium phosphate buffer for 45 min, rinsed in PBST, blocked with 0.5% bovine serum albumin in sodium phosphate buffer for 45 min and immunostained overnight with the primary rabbit anti-RFP (1:1000) and mouse anti-Prospero (1:100) antibodies in experiments verifying the *M{mira-His2A.mCherry.HA}* reporter. Alexa Fluor 568-conjugated goat anti-rabbit and Alexa Fluor 633-conjugated goat anti-mouse antibodies (1:1000) were used for secondary staining. In experiments to detect protein fucosylation, we used the same anti-RFP and anti-Pros primary antibodies in the primary incubation with Alexa Fluor 647-conjugated goat anti-HRP (1:500), Alexa Fluor 488-conjugated goat anti-mouse (1:1000) and Alexa Fluor 568-conjugated goat anti-rabbit (1:1000) antibodies in the secondary incubation. Antibodies for detecting Prospero were omitted in some experiments. After antibody incubations, samples were washed, counterstained with DAPI (1:10000) and mounted in Vectashield medium. Images were collected on a Leica SP8 scanning confocal microscope at the Indiana University Light Microscopy Imaging Center. Samples to be compared were acquired under the same settings and processed simultaneously using Adobe Photoshop CC. See Reagent Table (Table S1) for antibody sources.

### Transgene construction

*M{mira-His2A.mCherry.HA}* expresses sequences encoding a nuclear-localized *His2A* histone protein fused to a catamer of four mCherry coding sequences and the sequence for a C-terminal hemagglutinin (HA) tag. The *His2A* coding sequence is shared by several *His2A* gene repeats in the histone gene cluster. This fusion transcript is expressed under the control of *miranda* gene regulatory sequences (2.6 kb upstream and 1.6 kb downstream) that were previously shown to drive expression in intestinal progenitor cells (Bardin *et al.* 2010). The progenitor plasmid was generated using Gateway MultiSite cloning (Shearin *et al.* 2014) using plasmids provided by Steve Stowers (Montana State University). Full construction details are available upon request. It was inserted into the *M{3xP3-RFP.attP}ZH-2A* landing site by Rainbow Transgenic Flies, Inc. (Camarillo CA). The *loxP*-flanked *3xP3-RFP* cassette was subsequently removed from the progenitor insertion by Cre-mediated *in vivo* excision to leave only the *miniwhite* marker associated with the inserted sequence.

### High molecular weight DNA extraction for Nanopore sequencing

High molecular weight DNA extractions were performed as previously described with slight modifications to improve DNA yield (Miller *et al.* 2018b). A total of 40 male and female flies from Bloomington stock 2475 (w*; *T(2;3)ap*^*Xa*^, *ap*^*Xa*^*/In(2L)Cy*, *In(2R)Cy*, *Duox*^*Cy*^; *TM3*, *Sb*^*1*^) were collected, starved for 48 hr, and frozen at –80° for 72 hr before extraction. Frozen flies were transferred to a 2 mL Kimble Dounce homogenizer with 1 mL of homogenization buffer (0.1 M NaCl, 30 mM Tris-HCl pH 8.0, 10 mM EDTA, 0.5% Triton X-100) and homogenized with 10 strokes of looser-fitting pestle A and then 10 strokes of tighter-fitting pestle B. This homogenate was transferred to a 1.5 mL Eppendorf tube. The Dounce homogenizer was rinsed with an additional 500 µL homogenization buffer, and this was combined with the rest of the homogenate. This tube was centrifuged at 2,000 G for five minutes to pellet fly material. The supernatant was discarded and the pellet was resuspended in 100 µL of homogenization buffer using a wide-bore tip.

A fresh 1.5 mL Eppendorf tube was prepared with 380 µL of lysis buffer (0.1 M Tris-HCl pH 8.0, 0.1 M NaCl, 20 mM EDTA), 10 µL of Proteinase K (20 mg/mL), 10 µL 10% w/v SDS, and 5 µL RNAse A (Sigma R6148-1.7mL). Using a wide-bore tip, resuspended homogenate was transferred to this tube and mixed by pipetting. Lysis occurred at 50° for 6 hr, with gentle swirling and inversion every 45–60 min. If visible clumps of homogenate could not be broken up by gentle mixing, the tube was shaken briefly to encourage mixing.

Lysate was extracted twice with an equal volume (∼600 µL) of phenol/chloroform/isoamyl alcohol (25:24:1, pH 8.0) in a single 2 mL 5PRIME phase lock gel light tube. The extraction was mixed on a platform rocker at medium speed for eight minutes and then centrifuged for eight minutes at 16,000 G. To maximize DNA purity, the aqueous (upper) phase was extracted twice in the same tube. After the second extraction, the aqueous phase was carefully decanted into a new 2 mL phase lock gel tube and extracted once with an equal volume (∼600 µL) of chloroform as described above. The aqueous phase was then decanted quickly into a 2 mL Eppendorf DNA LoBind tube. DNA was precipitated by adding 0.1 volume of NaOAc, gently swirling to mix, and then adding 2.1 volumes of absolute ethanol. Gentle mixing by inversion was performed until all shimmering strands were precipitated into a white, stringy clump of DNA.

The DNA clump was transferred into a fresh 1.5 mL DNA LoBind tube with a wide bore tip. Excess ethanol was removed by pipetting, and the DNA pellet washed twice with 200 µL of 70% ethanol without centrifugation. Another 200 µL of 70% ethanol was added to the tube and the DNA was pelleted by centrifugation at 2,000 G for two minutes. All ethanol was removed, and the DNA dried for 5–10 min to the immediate moment where the pellet became translucent. Then, 100µL of TE pH 8.0 was added and the tube incubated at 50° for one hour, briefly spun in a tabletop centrifuge, and then incubated at 4° for 48 hr. DNA was lightly sheared by gently pipetting ten times through a P1000 tip and incubated at 4° for an additional 48 hr.

### Nanopore library preparation and sequencing

Nanopore libraries were prepared with the SQK-LSK109 Ligation Sequencing Kit with slight modifications to the standard protocol. To start the protocol, three micrograms of high molecular weight DNA were diluted with water to a total volume of 47.5 µL. The DNA repair and dA-tailing steps were then performed with a mixture of 47.5 µL sample, 3.5 µL FFPE DNA Repair Buffer, 2 µL FFPE DNA Repair Mix, 3.5 µL Ultra II End-prep Reaction Buffer, 3 µL Ultra II End-prep Enzyme Mix, and 0.5 µL 100x NAD+. This mixture was incubated in a 200 µL PCR tube at 20° for 60 min and 65° for 30 min in a thermal cycler, and then transferred to a 1.5 mL DNA LoBind tube. Because magnetic beads cause DNA clumping when the sample contains many large fragments, the sample was cleaned up without beads by adding an equal volume (60 µL) of precipitation buffer (9% w/v PEG 8000, 900 mM NaCl, 10 mM Tris-HCl pH 8.0), incubating for 15 min, and centrifuging for 30 min at 10,000 G. To wash the pellet, the supernatant was removed and 150 µL of SFB (from the kit) was added, and then centrifuged at 10,000 G for 2 min. The pellet was washed another time in this manner, and then immediately resuspended in 30 µL of 10 mM Tris-HCl pH 8.0. The sample was incubated at 50° for one hour, spun on a benchtop centrifuge briefly, and further resuspended at 4° for 48 hr.

For adapter ligation, half the volume recommended by the manufacturer’s protocol were used: 12.5 µL ligation buffer (LNB from the ligation kit), 5 µL T4 ligase, and 2.5 µL adapter mix (AMX from the ligation kit) was added to the 1.5 µL DNA LoBind tube containing the sample. After mixing, the sample was incubated for 30 min at room temperature. For the same reason as described previously, magnetic beads were omitted from the protocol. Since the ligation buffer causes DNA precipitation, the sample was centrifuged at 10,000 G for 30 min to pellet the DNA. To wash the pellet, the supernatant was removed and 150 µL LFB (from the ligation kit) added to the tube and centrifuged at 10,000 G for two minutes. The pellet was washed another time in this manner and immediately resuspended in 30 µL of 10 mM Tris-HCl pH 8.0. The sample was incubated at 40° for one hour, briefly spun down in a benchtop centrifuge, and stored at 4° for 48 hr. Small DNA fragments were removed from the prepared library using the Short Read Eliminator buffer (Circulomics) according to the manufacturer’s protocol, importantly, substituting LFB from the ligation kit or a 1:1 dilution of water and precipitation buffer instead of 70% ethanol to wash the pellet.

The adapter-ligated and size selected library was prepared for loading by quantifying with a Qubit fluorimeter and by transferring 350 ng (∼7.5 µL) of prepared library to a fresh 1.5 µL DNA LoBind tube. An equal volume (∼7.5 µL) of sequencing buffer (SQB from the ligation kit) was then added. Flush buffer (FB) from the EXP-FLP002 Flow Cell Priming Kit was added to a final volume of 70 µL. The library was loaded and sequenced according to manufacturer’s instructions. After 12 hr, the sequencing run was paused, and the flow cell was flushed with the EXP-WSH003 Flow Cell Wash Kit. A fresh library was loaded as described above and this procedure was performed one more time during the sequencing run.

Raw Nanopore reads were converted to FASTQ files using Guppy 3.2.4 in high-accuracy mode and all reads were aligned to release 6 of the *D. melanogaster* genome assembly using BWA MEM version 0.7.17-r1188 (Li and Durbin 2009).

### Sequencing of the Df(2L)bhe chromosome

Three pools of males heterozygous for the *Df(2L)bhe* chromosome from Bloomington stock 3268 and the second chromosome from the genome reference strain (Bloomington stock 2057) were collected from three separate single-male crosses to take into account cryptic genetic changes that have accumulated in the reference strain (Gutzwiller *et al.* 2015). DNA for sequencing was prepared as described in Miller *et al.* (2018a). Libraries were sequenced in 150-bp paired-end mode on an Illumina NextSeq 500 sequencer. Illumina Real Time Analysis version 2.4.6 was used to demultiplex reads and generate FASTQ files. Alignment to version 6 of the *Drosophila melanogaster* genome assembly was performed with bwa version 0.7.15-r1140 (Li and Durbin 2009) and SNPs were called using SAMtools version 1.5 (Li *et al.* 2009).

### Identification and analysis of shared and unique SNPs on *X* and third chromosome balancers

SNPs and indels for the *X* chromosome balancers *FM7a* and *FM7c* and the third chromosome balancer *TM3* were obtained from previous alignments (Miller *et al.* 2016a, 2016b). Polymorphisms with VCF quality scores greater than 220 were identified using VCFtools version 0.0.15 (Danecek *et al.* 2011) and then merged using vcf-merge. VCF files were annotated using SnpEff version 4.3 (Cingolani *et al.* 2012) and annotated files were filtered using custom scripts.

### Data availability

The accompanying tables contain complete complementation data. Stocks may be obtained from the Bloomington *Drosophila* Stock Center as indicated in the Reagent Table (Table S1). Basecalled Nanopore data are available from the National Center for Biotechnology Information (https://www.ncbi.nlm.nih.gov/) under project number PRJNA623115. Data related to characterization of the *Df(2L)bhe* chromosome are available under project PRJNA623116. Original data underlying this manuscript can be accessed from the Stowers Original Data Repository at http://www.stowers.org/research/publications/LIBPB-1520. Custom scripts used for data analysis, including the genome assembly of Bloomington stock 2475, are available on GitHub (https://github.com/danrdanny/balancerPhenotypes). Supplemental material is available at figshare: https://doi.org/10.25387/g3.12996710.

## Results

### Recessive lethal, female-sterile and visible phenotypes associated with third chromosome balancer breakpoints

The molecular analysis of third chromosome balancers presented by Miller *et al.* (2016a) and summarized in Table S2 raised the question of how the disruption of genes by inversion breakpoints contributes to the homozygous lethality of the balancers. Some component inversions were known to be homozygous viable and fertile, but some inversion breakpoints had not, to our knowledge, been examined. As shown in Tables 1 and S3, we tested all *TM3*, *TM6* and *TM6B* breakpoints for strong phenotypic effects by performing complementation tests with deficiencies spanning these breakpoints and scoring for lethality, female sterility or grossly abnormal morphology. For each test, we had independent control crosses to show the stocks were not compromised (Table S4).

**Table 1:** Assessing phenotypes associated with third chromosome balancer breakpoints

Inversion*^a^*	Balancer	Breakpoint*^b^*	Genes disrupted	Deletion or mutation tested	Phenotypes	Related observations
*In(3LR)P88*	*TM6*	61A6	Between *CG13485* & *CG12483*	*Df(3L)ED50002*, *Df(3L)ED4079*	Viable, female fertile	*Df(3L)ED50002* & *Df(3L)ED4079* are homozygous viable
		61B3	*Tudor-SN*	*Df(3L)BSC125*, *Df(3L)Exel6084*	Viable, female fertile	
		89B14	*ss*	*ss^1^*	Viable, female fertile, bristle loss	*ss* mutation associated with *In(3LR)P88* (Lindsley and Grell 1967; Morata and Lawrence 1978; Struhl 1982)
*In(3LR)HR33*	*TM6B*	61B2	Between *CG34453* & *E(bx)*	*Df(3L)ED201*, *Df(3L)Exel6084*	Viable, female fertile	*In(3LR)HR33* is homozygous viable (Ashburner 1972)
		87B3	Between *CG12256* & *CG3916*	*Df(3R)Exel7313*, *Df(3R)Exel6162*	Viable, female fertile	
*In(3L)P*	*TM6*, *TM6B*	63B9	*CG14964*	*Df(3L)BSC671*, *Df(3L)BSC672**^c^*	Viable, weak female fertility	*In(3L)P* is homozygous viable and fertile (Payne 1918, 1924).
		72E1	Between *CG13042* & *CR32160*	*Df(3L)BSC560*, *Df(3L)BSC579*	Viable, female fertile	
*In(3LR)sep*	*TM3*	65D3	Between *Prat2* & *CG45413*	*Df(3L)Exel6109*, *vvl^sep^*	Viable, female fertile, vein defects	*In(3LR)sep* homozygotes and other *vvl* mutants show vein defects (Diaz-Benjumea and García-Bellido 1990)
		85F2	*Glut4EF*	*Df(3R)Exel6155*, *Df(3R)Exel6154*, *Glut4EF^sep^*	Viable, female fertile, outheld wings	*Glut4EF* mutants have outheld wings (Yazdani *et al.* 2008)
*In(3LR)TM3-3*	*TM3*	71B6	*FucTA*	*Df(3L)ED218*, *Df(3L)BSC837*	Viable, weakly female fertile	*FucTA* mutants viable (Yamamoto-Hino *et al.* 2010)
		94D10	*p53*	*Df(3R)BSC803*, *Df(3R)ED6103*	Viable, female fertile	*p53* mutants viable and fertile (Rong *et al.* 2002; Sogame *et al.* 2003; Xie and Golic 2004)
*In(3LR)M6*	*TM6*, *TM6B*	75D6	*CR43987*	*Df(3L)BSC832*, *Df(3L)BSC416*	Viable, female fertile	Some *TM6C* chromosomes, which carry *In(3LR)M6*, are homozygous viable and fertile
		94A11	*CG13857*	*Df(3R)BSC511*, *Df(3R)BSC685*	Viable, female fertile	
*In(3LR)TM3-2*	*TM3*	76B1–2	*CG32206* & possibly *ms(3)76Ba*	*Df(3L)ED228*	Viable, female fertile, male fertile	
		92F4	*Lrrk*	*Df(3R)BSC141*, *Df(3R)BSC488*	Viable, female fertile, normal locomotion	Locomotory and female fertility loss in aging *Lrrk* mutants (Lee *et al.* 2007). We saw no overt phenotypes in young flies.
*In(3LR)TM3-1*	*TM3*	79F3	*CG14459*	*Df(3L)BSC451*, *Df(3L)ED230*	Viable, female fertile	
		100D1	*kek6*	*Df(3R)ED6361*, *Df(3R)BSC505*	Viable, female fertile	
*In(3R)Hu*	*TM6B*	84B2	Between *CR44933* & *Sodh-1*	*Df(3R)Antp17*	Viable, female fertile, malformed tergites	*In(3R)Hu* is homozygous viable (Bridges and Brehme 1944). *TkR86C* mutations are homozygous viable and fertile (Asahina *et al.* 2014)
		84F8	*CR44318*	*Df(3R)Exel6147*	Viable, female fertile	
		86C5	*TkR86C*	*Df(3R)BSC529*, *Df(3R)Exel6159*	Viable, female fertile	
*In(3R)C*	*TM3*, *TM6*, *TM6B*	92E2	Between *CG4362* & *CG42668*	*Df(3R)ED6025*, *Df(3R)ED6027*	Viable, female fertile	*In(3R)C* is homozygous viable and fertile (Dexter 1914; Muller 1918; Bridges and Morgan 1923; Sturtevant 1926)
		100F2–3	Distal to genes	*Df(3R)ED50003*, *Df(3R)ED6361*, *Df(3R)ED6362**^d^*	Viable, female fertile	

a The *In(3LR)TM3-1*, *In(3LR)TM3-2* and *In(3LR)TM3-3* inversions are named here for the first time.

b Cytological bands predicted from sequence coordinates (Table S2) using FlyBase reference table except 100F2–3, where we cite polytene analysis (Morgan *et al.* 1937)

c Fertility higher in *TM6B* crosses than *TM6* crosses with both deletions.

d *P{RS3}CB-0686-3*, the FRT-bearing progenitor of the distal *Df(3R)ED6361* and *Df(3R)ED6362* breakpoints, lies within the subtelomeric region (Phalke *et al.* 2009) at a position that may not be distal to the *In(3R)C* breakpoint.

We verified that some inversion breakpoints are associated with recessive visible phenotypes (Table 1). The 89C breakpoint of *In(3LR)P88* lies in the fourth intron of *spineless* (*ss*), resulting in short bristles (Duncan *et al.* 1998). *In(3LR)sep* failed to complement alleles of *ventral veins lacking* (*vvl*) and deletions of the region of the 65D breakpoint, resulting in wing venation defects (Diaz-Benjumea and García-Bellido 1990; de Celis *et al.* 1995). Third chromosome balancers do not appear to contain mutations in the *vvl* coding region (Miller *et al.* 2016a), so disruption of *vvl* function by the *In(3LR)sep* breakpoint ∼130 kb downstream of *vvl* likely comes from perturbing long-distance regulation. The 85F breakpoint of *In(3LR)sep* disrupts *Glut4EF*, resulting in outspread wings (Yazdani *et al.* 2008; Miller *et al.* 2016a). *Df(3R)Exel6154*, which deletes only a subset of *Glut4EF* 5′ exons, showed phenotypes one would expect of a hypomorphic *Glut4EF* allele. Unlike flies with amorphic *Glut4EF* genotypes, which showed obvious wing spreading soon after eclosion and progressed to strong spreading within a week, flies with mutant genotypes involving *Df(3R)Exel6154* showed infrequent and slight spreading in the first three days after eclosion and progressed to intermediate frequency and severity by one week (Tables 1 and S3).

We were surprised to find that none of the breakpoints were associated with complete lethality or female sterility (Table 1). We did not measure viability or fertility rates in ways that would allow us, in most cases, to identify intermediate levels, but the 71B breakpoint on *TM3*, which disrupts the *FucTA* gene, was associated with severely reduced female fecundity. The relative innocuousness of the breakpoints likely reflects the care taken in choosing preexisting progenitor inversions to assure that balancers would be as trouble free as possible and in designing screens for isolating new inversions that did not rely on lethal or sterile phenotypes. These results suggest that the recessive lethality of third chromosome balancers in most current stocks is attributable to spontaneous mutations that have accumulated since the balancers were generated, although it is possible that deleterious effects of multiple breakpoints contribute to recessive lethality.

### A TM3 inversion breakpoint disrupting the p53 gene results in loss of apoptosis in response to irradiation

The 94D breakpoint of *In(3LR)TM3-3* on *TM3* lies within an intron of the *p53* gene. The exons on either side of this breakpoint encode a protein region involved in DNA binding in all *p53* isoforms, suggesting that the *p53* allele on *TM3* is amorphic. Previous studies have shown that homozygosity for *p53* null alleles does not result in lethality or sterility in Drosophila (Rong *et al.* 2002; Sogame *et al.* 2003; Xie and Golic 2004), so viability and fertility in our complementation tests of *TM3* and deletions of *p53* (Table 1) was not surprising. Nevertheless, other studies have shown that apoptotic responses to DNA damage are defective in *p53* mutants (Lee *et al.* 2003; Brodsky *et al.* 2004; Mehrotra *et al.* 2008; Qi and Calvi 2016).

**Figure 1:**
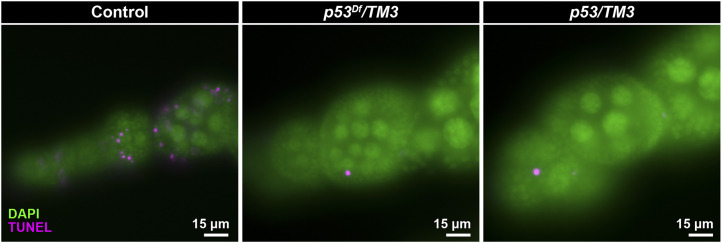
The *TM3* breakpoint at 94D disrupts *p53* apoptosis activity. Irradiation-induced apoptosis is seen as TUNEL staining in stage 1–5 follicle cells counterstained with DAPI of control females (*e.g.*, *y^1^ w^67c23^* homozygotes shown here). TUNEL staining is absent in follicle cells of females carrying *TM3* combined with chromosomal deletions removing the *p53* gene (*e.g.*, *TM3*/*Df(3R)ED6103* shown here) and females carrying *TM3* and loss-of-function *p53* alleles (*e.g.*, *TM3/p53*^*5A-1-4*^ shown here). TUNEL-stained cells in *p53* mutants are polar follicle cells, which undergo *p53*-independent, developmentally programmed cell death. See Table S5 for all genotypes tested.

To determine whether the *TM3* breakpoint eliminates *p53* activity, we examined the effect of the breakpoint on apoptosis in a convenient proliferative cell population, the mitotically dividing ovarian follicle cells that surround maturing egg chambers. Females with putative *p53* loss-of-function genotypes were irradiated, aged four hours and assayed for apoptosis by TUNEL assay. Unlike wild-type controls, which showed TUNEL staining in ∼12% of dividing follicle cells, the cells from females where *TM3* had been combined with a *p53* deficiency (*Df(3R)ED6103* or *Df(3R)BSC803*) or null mutation (*p53*^*5A-1-41*^ or *p53*^*-ns*^) had much lower frequencies of TUNEL staining (<1% of cells, a level similar to unirradiated controls; Figure 1, Table S5).

These results show that *p53* function is indeed disrupted by the *TM3* breakpoint and highlight the danger of balancing *p53* mutations with *TM3* and viewing the genotypes as “wild type”. A broader problem, however, is the effect of reducing *p53* copy number on cellular processes. *p53* is haploinsufficient with respect to the induction of apoptosis in response to telomere loss (Kurzhals *et al.* 2011) and may show haploinsufficiency with respect to other stress responses. Consequently, investigators should be careful not to use *TM3* heterozygotes as “normal” controls in experiments that might involve *p53*-related processes.

### A TM3 inversion breakpoint disrupts FucTA resulting in loss of immunostaining with antibodies against horseradish peroxidase

**Figure 2:**
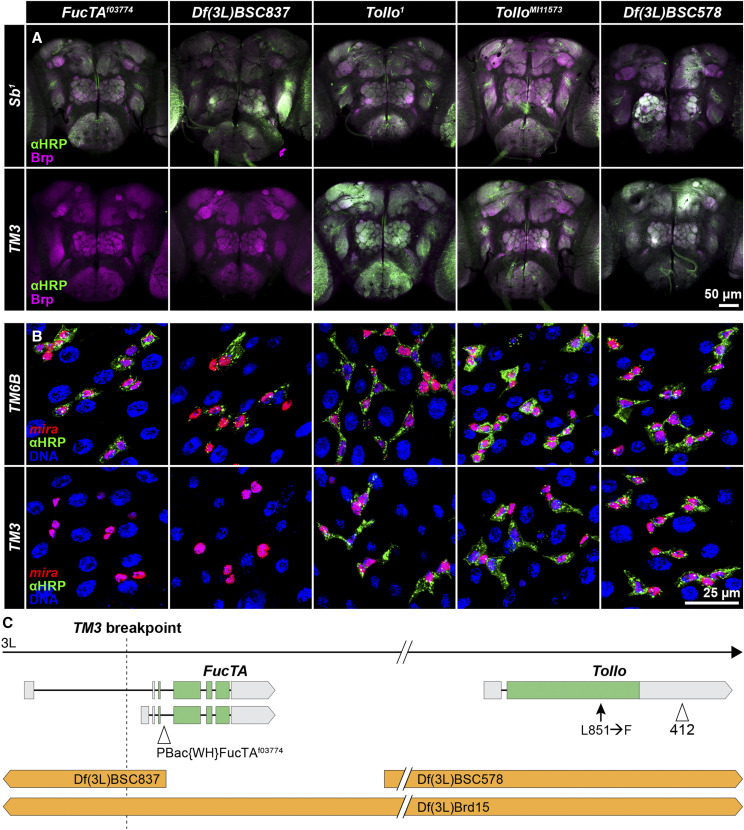
The *TM3* breakpoint in *FucTA* is associated with loss of anti-HRP antibody staining in nervous tissue and intestinal epithelial cells. A. *TM3*/*Sb*^*1*^ flies were crossed to mutation- or deletion-bearing flies. Anti-HRP staining was not detected in adult brains when deletions of *FucTA* (*Df(3L)BSC837* and *Df(3L)Brd15*) or a *FucTA* mutation (*FucTA*^*f03774*^) were combined with *TM3*, but it was seen in control crosses where a chromosome with no *FucTA* mutation (*Sb*^*1*^) was combined with the same deletions or mutation. Staining was seen with either *TM3* or *Sb*^*1*^ combined with a *Tollo* deletion (*Df(3L)BSC578*) or mutations (*Tollo*^*1*^ and *Tollo*^*MI11573*^). Anti-HRP staining shown in green; anti-BRP counterstaining shown in magenta to highlight neuropils. B. Crosses between *mira-His2A.mCherry.HA*; *wg^Sp-1^/CyO*; *TM3/TM6B* females and males carrying the same deletions or mutations gave identical results for anti-HRP staining of intestinal epithelial cells where *TM6B* serves as the *FucTA*^*+*^ control (and only *wg^Sp-1^/+* progeny were scored). Anti-HRP staining shown in green; *miranda*-expressing progenitor and enteroendocrine cells shown in red; DAPI staining of nuclei shown in blue. C. The 71B breakpoint in *TM3* lies within an alternative 5′ UTR of *FucTA* and ∼85 kb distal to *Tollo*. *Df(3L)Brd15* deletes both genes while *Df(3L)BSC837* disrupts only *FucTA* and *Df(3L)BSC578* deletes only *Tollo*. *FucTA*-specific mutations and deletions failed to complement *TM3* with respect to anti-HRP staining while *Tollo*-specific mutations and deletions complemented. A *412* transposon insertion in the 3′ UTR and a polymorphism leading to substitution of phenylalanine for leucine are present in *Tollo* in all *TM3* chromosomes examined, but they may be neutral with respect to *Tollo* function.

The 71B breakpoint of *In(3LR)TM3-3* on *TM3* lies within the 5′ UTR of one of two reported transcripts of the *Fucosyltransferase A* (*FucTA*) gene and likely results in partial loss of expression (Miller *et al.* 2016a). The *FucTA* enzyme catalyzes the attachment of fucose to N-linked glycans on a variety of proteins (Fabini *et al.* 2001). This modification is detected by antibodies raised against horseradish peroxidase (HRP) and is enriched in neural tissues of ecdysozoans including Drosophila (Jan and Jan 1982; Haase *et al.* 2001). Yamamoto-Hino *et al.* (2010) showed that labeled anti-HRP antibodies do not stain the nervous system of *FucTA*^*f03774*^ mutant larvae and Snow *et al.* (1987) demonstrated that neural staining is lost in homozygous *TM3* embryos (though a few nonneural tissues show staining). As expected, when we combined *TM3* from multiple stocks with either a deletion of the *FucTA* gene (*Df(3L)BSC837*) or *FucTA*^*f03774*^, we saw loss of anti-HRP staining in adult brains (Figure 2, Table S6).

To test whether *FucTA* mutations affect anti-HRP antibody staining in another tissue, we examined the intestine, where O’Brien *et al.* (2011) showed staining of progenitor cells (stem cells and enteroblasts). To facilitate this analysis, we generated a new cell marker containing regulatory sequences from the *miranda* gene previously shown to be expressed in progenitor cells (Bardin *et al.* 2010) and characterized its expression relative to known progenitor and enteroendocrine cell markers (Micchelli and Perrimon 2006). Figure S1 shows that it is expressed strongly in progenitor cells and weakly in some enteroendocrine cells in a pattern matching anti-HRP staining. As we saw in neurons, *TM3* combined with either *FucTA* deletions or *FucTA*^*f03774*^ eliminated anti-HRP intestinal cell staining (Figure 2; Table S6). This observation highlights the utility of *FucTA* mutations and anti-HRP antibody staining for investigating the significance of protein fucosylation in intestinal cells, which remains largely unexplored.

The association of fucosylation defects with disruption of *FucTA* by a *TM3* breakpoint would be straightforward were it not for previous studies suggesting that *Tollo*, a gene linked closely to *FucTA*, is disrupted on *TM3* chromosomes and that *Tollo* regulates fucosylation. Seppo *et al.* (2003) mapped loss of embryonic nervous system anti-HRP staining on *TM3* to the region of *Df(3L)Brd15*, showed that *412* transposon sequences exist within the 3′ UTR of *Tollo* on *TM3* (Figure 2C), and speculated that the *412* sequences coincided with the *TM3* breakpoint. Our long-read sequence showed that the *412* element is full length and not associated with an aberration breakpoint, but an examination of the short-read sequence data from Miller *et al.* (2016a) verified *412* termini in the same position (3L:15,241,752–15,241,755) in a broad sampling of *TM3* chromosomes. We also saw that all sequenced *TM3* chromosomes share a polymorphism in *Tollo* encoding a leucine-to-phenylalanine substitution at amino acid 851. We now know, however, that *Df(3L)Brd15* deletes both *FucTA* and *Tollo*, and that fucosylation defects that have been attributed to *Tollo* mutation may be the result of *FucTA* mutation. We did not see immunostaining eliminated in adults carrying *TM3* and *Tollo* mutations or *Df(3L)BSC578*, which deletes *Tollo* but leaves *FucTA* intact (Figure 2, Table S6), which is consistent with the results of Yagi *et al.* (2010), who saw immunostaining in embryos homozygous for the null *Tollo*^*59*^ mutation. We also saw immunostaining in *FucTA* heterozygotes when *Tollo* copy number was reduced (Table S6). While results suggest that the immunostaining defects are not attributable to *Tollo* disruption, Seppo *et al.* (2003) reported rescue of anti-HRP staining when they combined *Df(3L)Brd* and *TM3* with transgenic constructs expressing *Tollo*, Baas *et al.* (2011) reported loss of immunostaining in flies homozygous for the *Tollo*^*C5*^ null allele, and the position of the *TM3* breakpoint in the 5′ UTR of an alternative *FucTA* transcript may allow plastic expression. Reconciling these observations is beyond the scope of this paper, but our studies indicate that the purported regulation of fucosylation by *Tollo* should be reevaluated.

### Polymorphisms on FM7a, FM7c and TM3 balancers disrupt protein-coding genes

The second chromosome balancers *SM5*, *CyO* and *SM6a* have been shown to share mutations that affect protein-coding genes (Miller *et al.* 2018a). For example, all *SM5* and *SM6a* balancers carry a C-to-T mutation in the uncharacterized gene *CG12506*, resulting in a premature translational stop. In addition, every sequenced balancer has unique mutations that are not seen on any other balancer chromosome, indicating that spontaneous mutations occur in stocks at an appreciable frequency. We therefore undertook a similar analysis of previously sequenced *X* and third chromosome balancers (Miller *et al.* 2016a, 2016b) to identify SNP and indel polymorphisms creating missense, splice-site, or nonsense mutations that are either shared or unique among the *FM7a*, *FM7c* or *TM3* balancers (Table 2). We identified 1,536 mutations shared among all eight *FM7a* and *FM7c* balancers (Table S7), 339 mutations shared only among the three *FM7a* balancers sequenced (Table S8), 56 mutations shared only among all five *FM7c* balancers (Table S9) and 3,011 mutations shared among at least 15 of 17 *TM3* balancers (Table S10). Altogether, we found 1,722 mutations unique to individual balancer chromosomes that likely affect protein function including 1,652 missense mutations, 25 stop mutations, and 45 splice-site mutations (Table S11). These statistics highlight a challenge in using balancers experimentally: specific balancers carry unique constellations of polymorphisms that may affect experimental outcomes and the interpretation of results.

**Table 2:** Number of SNP or indel mutations shared by multiple balancers or present on only one balancer

Balancer	Stock*^a^*	Stop mutations	Missense mutations	Splice-site mutations
**Mutations on multiple balancers**				
*FM7a*	All	0	331	8
*FM7a and FM7c*	All	11	1503	22
*FM7c*	All	0	56	0
*TM3*	All	7	2956	48
**Mutations on a single balancer**				
*FM7a*	785	0	7	0
*FM7a*	35522	0	6	0
*FM7a*	36489	0	17	1
*FM7c*	616	0	4	2
*FM7c*	3378	0	7	3
*FM7c*	5193	1	114	2
*FM7c*	23229	0	34	0
*FM7c*	36337	0	70	3
*TM3*	120	1	50	3
*TM3*	500	0	33	3
*TM3*	504	3	265	1
*TM3*	560	6	419	8
*TM3*	1614	0	129	3
*TM3*	1679	1	7	0
*TM3*	2053	3	51	2
*TM3*	2098	0	16	1
*TM3*	2198	0	9	1
*TM3*	2485	0	17	0
*TM3*	3251	2	8	0
*TM3*	5457	0	7	2
*TM3*	8852	6	93	2
*TM3*	9013	0	29	0
*TM3*	22239	0	92	4
*TM3*	24759	0	66	0
*TM3*	38418	3	183	4

a These numbers identify the original balancer stocks (Table S1) outcrossed to a common stock for sequencing (Miller *et al.* 2016a, 2016b).

### An Ankyrin 2 mutation is present on Sb^1^-marked TM3 chromosomes

In a series of crosses involving mutations in polytene region 65D–F several years ago, we noticed that flies inheriting *TM3* and the deletions *Df(3R)RM5-1*, *Df(3R)RM5-2* or *Df(3R)pbl-X1* were largely lethal, but escapers had unexpanded wings, disarranged bristles, small body size, improperly tanned cuticle, dark pigmentation, low female fecundity and a generally weak and sickly appearance (Table S12). The partial lethality of *TM3* with *Df(3R)RM5-1* and *Df(3R)RM5-2* had been noted previously (Grasso *et al.* 1996). Our subsequent analysis of *TM3* breakpoints with molecularly defined deletions (Table 1) showed that the 65D2–3 breakpoint of *In(3LR)sep* is not associated with these abnormal phenotypes and indicated that they were instead attributable to a mutation immediately proximal to the breakpoint. We mapped the mutation to a seventeen-gene interval based on *Df(3R)RM5-1* deleting *liquid facets* through *Ankyrin 2* (*Ank2*) (Koch *et al.* 2008). *TM3* sequences (Miller *et al.* 2016a) showed the presence of a deletion within the last exon of most *Ank2* transcripts that removes thirteen nucleotides (*3L*:7,658,562–7,658,574) and affects all but three *Ank2* isoforms. A transposon insertion into the same exon, *PBac{WH}Ank2*^*f02001*^, produced the same phenotypes in combination with *TM3* (Table S12), consistent with the report of Koch *et al.* (2008) that most *Ank2* mutations could not be maintained in stock using *TM3*.

Interestingly, it appears from the *TM3* sequences (Miller *et al.* 2016a) and complementation tests against *Ank2* deletions and *Ank2*^*f02001*^ (Table S12) that all *TM3* chromosomes marked with *Sb*^*1*^ (or chromosomes derived from them) carry the *Ank2* mutation. The original version of *TM3* lacking the *Sb*^*1*^ and *Ser*^*1*^ markers and a later version carrying only *Ser*^*1*^ that was the immediate progenitor of all *Sb*^*1*^-marked *TM3* chromosomes (Tinderholt 1960; Miller *et al.* 2016a) lack the *Ank2* mutation. It seems likely that the *Ank2* mutation, which we call *Ank2*^*TM3*^, arose spontaneously on *TM3* around the time *Sb*^*1*^ was introduced and has since been propagated to all *Sb*^*1*^-marked *TM3* balancers.

### The distal breakpoint of In(3R)C lies between subtelomeric heterochromatin and the telomeric transposon array

A previous analysis of third chromosome balancers (Miller *et al.* 2016a) was unable to determine the precise genomic positions of the *In(3R)C* breakpoints using short-read sequencing, because the distal breakpoint lies within repetitive sequence at the distal end of chromosome arm 3R. Recently, chromatin conformation capture data from *TM3* heterozygotes was used to estimate the genomic position of the proximal 92E breakpoint to 3R:20,308,200 (Ghavi-Helm *et al.* 2019). Using this estimate as a guide, we isolated long Nanopore sequencing reads that spanned the proximal and distal *In(3R)C* breakpoints and performed a *de novo* genome assembly using Flye (version 2.7-b1585) with default parameters and the “–keep-haplotypes” flag (Kolmogorov *et al.* 2019).

**Figure 3:**
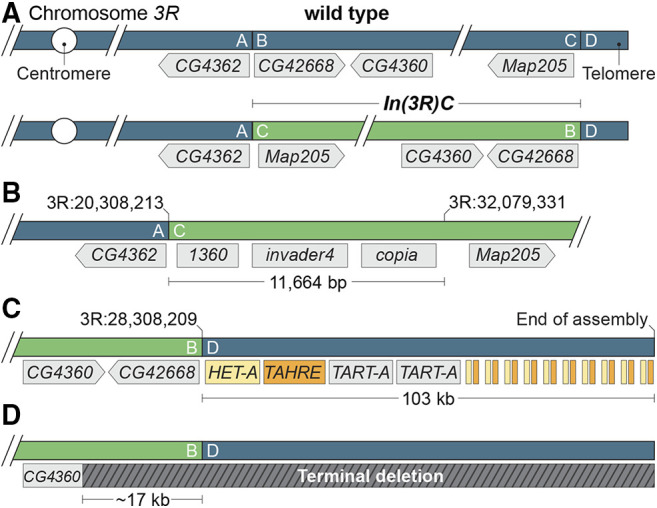
The structure of *In(3R)C*. A. The wild-type and inverted arrangement of the *In(3R)C* inversion breakpoints with neighboring genes labeled. The exact position of the distal (C|D) breakpoint was not previously known, but was suspected to lie within subtelomeric heterochromatin. B. Assembly of long sequencing reads revealed the molecular structure of the proximal (A|C) junction, with nearly 12 kb of sequence between the distal end of the reference *3R* assembly at 32,079,331 and the distal break. This region contained three transposable elements that were, in all likelihood, originally positioned immediately proximal to the telomeric transposon repeats. C. The distal *In(3R)C* breakpoint fell immediately proximal to telomeric repeats. Our assembly of this region extended ∼103 kb distally from the distal (B|D) junction and contained the *HeT-A*, *TAHRE*, and *TART-A* elements expected for a telomeric region as well as repeated fragments of *HeT-A* and *TART-A* at the distal end of the assembly. The positions and number of elements shown distal to the second *TART-A* element are estimates. All elements, including the incomplete *HeT-A* and *TAHRE* fragments, are oriented with their 3′ ends toward the centromere. D. Having established the structure of *In(3R)C*, we interpret the ∼17 kb deletion of sequences immediately distal to 3R:20,308,209–20,308,213 observed by Ghavi-Helm *et al.* (2019) as evidence of a *3R* terminal deletion specific to the *TM3* chromosome they characterized.

We localized the proximal *In(3R)C* breakpoint to 3R:20,308,209–20,308,213 (the breakpoint contains a 5 bp duplication) in the intergenic region between *CG4362* and *CG42668* (Figure 3). The assembly spanning the proximal inversion end (shown as the A|C junction in Figure 3A) juxtaposed sequences distal to *CG4362* to sequences distal to *Map205*, the distalmost gene on 3R. This assembly included the distalmost sequences of the reference genome assembly at 32,079,330 and 11,664 bp of novel sequence distal to it, which contained *1360*, *invader4* and *copia* transposons (Figure 3B, File S1). These transposons are commonly found in subtelomeric regions (Anderson *et al.* 2008; Mason and Villasante 2013).

The distal end of *In(3R)C* (shown as the B|D junction in Figure 3A) juxtaposes sequences immediately proximal to *CG42668* to ∼103 kb of sequence composed of telomeric *HeT-A*, *TAHRE*, and *TART-A* transposons adjacent to repeated partial fragments of *HeT-A* and *TAHRE* (Figure 3C). To confirm this assembly, we identified individual reads that spanned the inversion end and extended up to 50 kb distally and found that they included the same *HeT-A*, *TAHRE*, and *TART-A* transposons. No individual read extended past the first *TART-A* element, so we could not confirm the presence of the second *TART-A* element or the subsequent *HeT-A* and *TAHRE* fragments with this approach. The repeated *HeT-A* and *TAHRE* fragments are similar in structure to partial duplications of *HeT-A* and *TART* elements observed by Levis *et al.* within telomeric sequences (Levis *et al.* 1993). The nongenic positions of the breakpoints largely explain why *In(3R)C* gives no overt phenotypes when homozygous (Dexter 1914; Muller 1918; Bridges and Morgan 1923; Sturtevant 1926) or combined with deletions for the regions of the breakpoints (Table 1). These complementation tests provided no evidence for position-effect suppression of genes juxtaposed to telomeric or subtelomeric sequences by *In(3R)C*.

Our results show that the distal *In(3R)C* breakpoint separates telomere-associated sequences (TAS) from telomeric sequences, and moves them from their usual subtelomeric position. While the two domains are juxtaposed on most chromosomes (Asif-Laidin *et al.* 2017), their relationship is unclear. Both express piRNAs to repress transposon activity, but the domains are regulated differently in germ line and somatic cells (Radion *et al.* 2018). TAS regions have heterochromatic properties that may be relevant to telomere function (Radion *et al.* 2018), but TAS are absent from some chromosomes (Asif-Laidin *et al.* 2017) and *In(3R)C* has a worldwide distribution in wild populations (Inoue and Igarashi 1994). These observations indicate that any interdependence of the two domains is complicated and suggest *In(3R)C* may prove valuable in exploring their interactions.

As discussed previously (Miller *et al.* 2016a, 2018a), meiotic recombination in chromosomal regions distal to the distalmost balancer breakpoint may be relatively high if the balancer breakpoint is positioned a large distance from the telomere, *e.g.*, the 65D breakpoint of *TM3*. In this context, the distal 3R breakpoint of *In(3R)C* provides the “perfect” balancer end, because no genes lie distal to the breakpoint. Consequently, even extremely distal 3R mutations can be maintained with confidence in stocks utilizing the *In(3R)C*-containing balancers *TM3*, *TM6*, *TM6B*, *TM6C*, *TM8* and *TM9*.

Interestingly, the chromatin conformation capture data of Ghavi-Helm *et al.* (2019) contained a ∼17-kb deletion immediately distal to the 92E breakpoint of *In(3R)C* (region B in Figure 3), which we did not see in our long-read sequencing data or on reanalysis of seventeen previously sequenced *TM3* chromosomes. This observation indicates that the *TM3* balancer they used in their studies contained a terminal deletion that removed the *3R* tip and euchromatic sequences placed near the tip by the inversion event (Figure 3D). So, while *CG42668* and the adjacent *CG4360* gene were intact in the seventeen *TM3* chromosomes sequenced previously and the *TM3* chromosome sequenced in this study, they were deleted in the *TM3* balancer analyzed by Ghavi-Helm and colleagues. We do not know if *Het-A*, *TAHRE* or *TART* elements have transposed to the truncated end of the balancer sequenced in their study. Unrecognized terminal deletions are not unusual in Drosophila. For example, cryptic *2L* terminal deletions removing the *l(2)gl* tumor suppressor gene have bedeviled studies of growth control (Roegiers *et al.* 2009). These results show that terminal deletions are yet one more kind of genetic variation involving balancers that can affect experimental outcomes.

### In(2L)Cy arose by ectopic recombination Between transposon insertions

**Figure 4:**
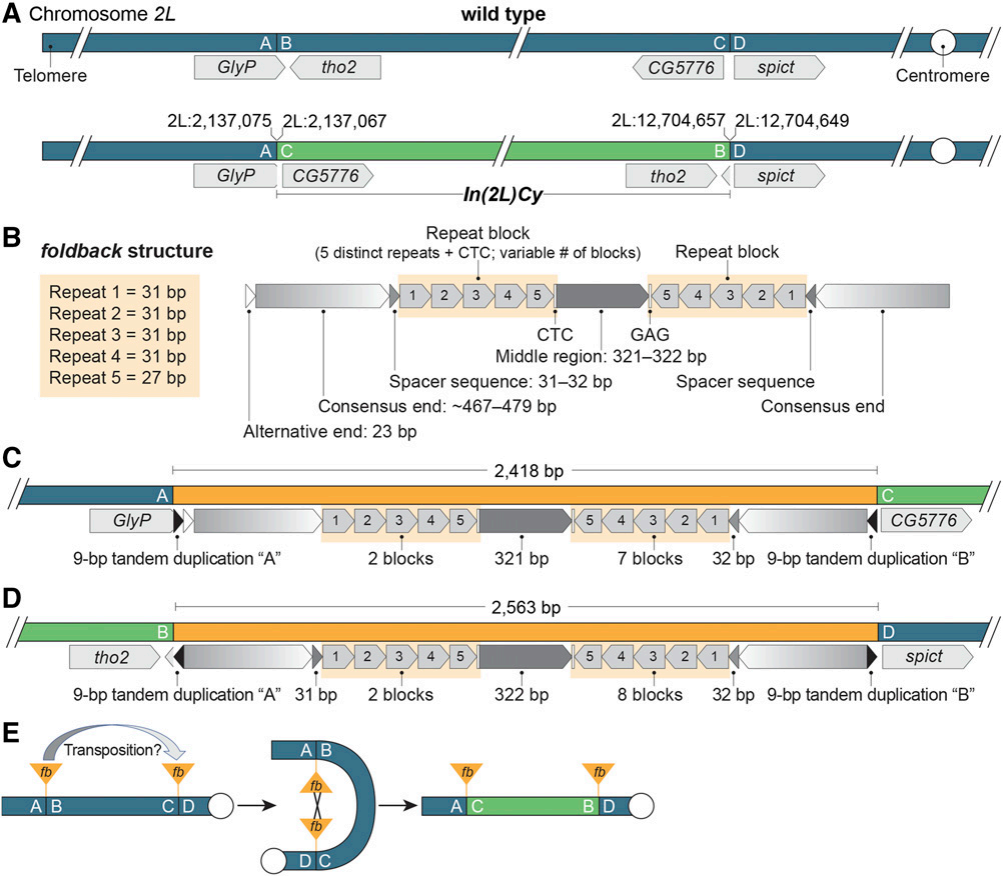
*In(2L)Cy* was likely created by ectopic recombination between two *foldback* transposable elements. A. The distal *In(2L)Cy* breakpoint (A|B) lies in the 3′ UTR of *GlyP* and proximal breakpoint (C|D) lies between *CG5776* and *spict*. Reference genome coordinates are shown. B. The general structure of the breakpoint-associated *FB* insertions. Both insertions have end sequences, spacer sequences and blocks of repeats in mirrored orientations flanking a middle region. Each block contains a single copy of five distinct short repeats, blocks are repeated tandemly a variable number of times, and each block terminates with a CTC motif. An additional 23 bp may be appended to a consensus end sequence. C. The distal (A|C) end of *In(2L)Cy* includes 2,418 bp of *FB* sequences, which contain an alternative end sequence and clusters of two and seven repeat blocks, but lack one spacer sequence. Consistent with a recombinant origin, the *FB* sequences are flanked by a 9 bp tandem site duplication from *FB* transposition into *GlyP* (shown as “A”) and another from *FB* transposition into the *CG5776**–**spict* region (“B”). D. The proximal (B|D) end includes 2,563 bp of *FB* sequences including two spacer sequences and clusters of two and eight repeat blocks. These *FB* sequences are flanked by the 9 bp duplicated sequences from *FB* transposition into *GlyP* (“A”) and the *CG5776**–**spict* region (“B”) expected for inversion via ectopic recombination. E. *In(2L)Cy* arose by recombination between progenitor *FB* insertions. The high similarity of the *FB* sequences at the *In(2L)Cy* ends and the presence of an alternative end sequence at the distal *In(2L)Cy* end suggests that an *FB* element within *GlyP* transposed to the *CG5776**–**spict* region and subsequently recombined with an *FB* element at the donor site.

Similar to *In(3R)C* discussed above, Miller *et al.* (2018a) were unable to localize the breakpoints of *In(2L)Cy*, a component of most second chromosome balancers, with single-nucleotide resolution using short sequence reads due to the presence of repetitive sequences at the breakpoints. Ghavi-Helm *et al.* (2019) used chromatin conformation capture data to estimate the positions of these breakpoints followed by paired-end sequencing to provide precise coordinates. To confirm their breakpoint mapping and provide more details about the inversion event, we sequenced DNA from flies carrying *In(2L)Cy* using long-read sequencing and identified multiple reads spanning both the distal and proximal *In(2L)Cy* breakpoints. We confirmed that the distal breakpoint lies at 2L:2,137,067–2,137,075 in the 3′ UTR of *GlyP* and the proximal breakpoint lies at 2L:12,704,649–12,704,657 in the intergenic region between *CG5776* and *spict* (Figure 4A). These breakpoints have no seriously deleterious effects: *In(2L)Cy* has no overt phenotypic effects when homozygous (Sturtevant 1931) or when combined with deletions for the breakpoint regions (the deletions tested by Miller *et al.* (2018a) span the refined breakpoint positions reported here).

From the *de novo* assembly produced by Flye and the sequences of individual long reads, we determined that each *In(2L)Cy* breakpoint is associated with an intact *FB* transposon that shows high conservation with *FB* elements characterized previously (Badal *et al.* 2006b). Each *FB* element carries inverted repeat end sequences 467 to 479 bp in length (Figure 4B) and the four end sequences showed ≥97% identity (File S1). Internal to the end sequences and flanking a middle region, the *FB* elements carry a single spacer region and a variable number of blocks of five 27–31 bp repeat sequences oriented in opposite directions. Both *FB* elements had two blocks of repeats distal to the middle region, but the distal *FB* element had seven blocks proximal to the middle region (Figure 4C) while the proximal *FB* element had eight (Figure 4D). All five repeats within all blocks showed high identity to consensus sequences and all repeat blocks were separated by single CTC motifs as expected (Badal *et al.* 2006b). The middle regions of *FB* elements often show some conservation, but the 321–322 bp middle regions of the *In(2L)Cy* breakpoint *FB* elements were essentially identical (99% identity) and contained three degenerated vestiges of two different 31-bp repeat sequences. No *NOF* transposons were present, even though they are often inserted into *FB* elements (Badal *et al.* 2006b, 2013).

That both breakpoints are associated with *FB* transposons strongly suggests that *In(2L)Cy* arose by ectopic recombination between transposon insertions, a major mode by which inversions arise in Drosophila populations (Ranz *et al.* 2007; Delprat *et al.* 2009; Reis *et al.* 2018; Orengo *et al.* 2019). The presence of end, spacer and block repeats makes *FB* and *FB*-like elements particularly recombinogenic (Cáceres *et al.* 1999; Casals *et al.* 2003; Moschetti *et al.* 2004; Badal *et al.* 2006a; Delprat *et al.* 2009) and the mirrored orientations of repeats allow single exchange events to produce inversions regardless of the relative orientations of *FB* insertions on progenitor chromosomes (Figure 4E). Because we do not know the sequences of the progenitor insertions, we cannot localize the sites of exchange within the *FB* elements.

The high sequence identity of the middle regions and the similarity in numbers and arrangements of repeat blocks make the two *FB* elements associated with *In(2L)Cy* look more like each other than any other *FB* element we have identified in any sequenced genome. This observation suggests that transposition of an *FB* element to a new site was followed by ectopic recombination with the donor site to invert the intervening chromosomal segment (Figure 4E). The difference in the number of repeat blocks (seven *vs.* eight), the absence of one spacer sequence and the minor differences in repeat sequences could easily have arisen, in the time since, by unequal sister chromatid exchange and spontaneous mutation—and larger changes, such as the repeat-mediated inversion of sequences within *FB* elements, are conceivable. In this scenario, the insertion in *GlyP* was likely the donor, because one *FB* end is distinctive: there are 23 bp of sequence between the conserved *FB* end sequence and the 9 bp tandem duplication generated upon *FB* insertion. This 23 bp sequence is also associated with an *FB* end in the reference genome sequence (*FB{}nmo^FB^*), suggesting that alternative end sequences may occasionally be used in transposition. The conserved end lying internal to the 23 bp sequence in the *GlyP* insertion would have been available for *FB* transposition to the *CG5776*–*spict* intergenic region. The alternative, that the progenitor insertions arose from independent transpositions of very closely related *FB* elements, is also possible. *FB* elements quite similar to those associated with *In(2L)Cy* do exist, even though they appear to be uncommon, *e.g.*, there are two *FB* insertions in the reference genome sequence (*FB{}CG34376^FB^* and *FB{}773*) with identical middle regions (≥99% identity among the four *FB* insertions) even though they differ in the number and arrangement of repeat blocks.

### Recessive lethal or sterile mutations in extremely distal positions add balancing power

**Figure 5:**
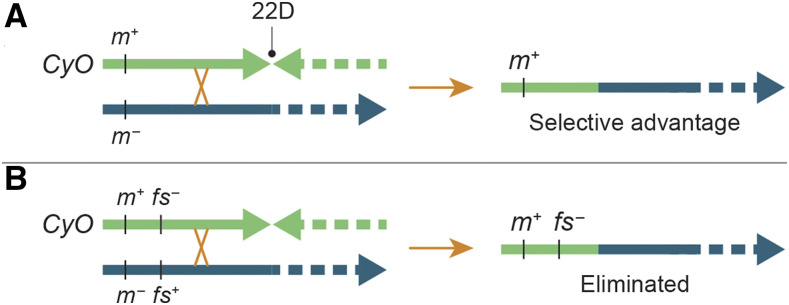
A deleterious mutation can prevent stock breakdown when a crossover occurs distal to the distalmost balancer breakpoint. Panel A shows that a meiotic crossover distal to the *CyO* breakpoint in region 22D can result in the formation of mutation-free recombinant chromosomes, which will have a selective advantage in a stock population. In contrast, panel B shows that the addition of a recessive lethal or sterile mutation (shown here as a female-sterile mutation) to the balancer leads to elimination of the same recombinant chromosomes through routine population dynamics.

Miller *et al.* (2018a) showed that *CyO* balances distal 2L mutations poorly because single crossovers occur distal to the distalmost breakpoint in 22D at notable frequency to give rise to nonbalancer chromosomes lacking distal mutations (Figure 5A). We were therefore puzzled by the unusual stability of a *CyO* stock carrying the *broadhead* mutation, which Kahsai and Cook (2018) showed is associated with a small terminal deletion called *Df(2L)bhe*. This mutation, which affects development of the anterior end of homozygous embryos (Nüsslein-Volhard *et al.* 1984; Tearle and Nüsslein-Volhard 1987), has been maintained stably in stock with *CyO* for approximately 40 years. Our short-read sequencing data from a sample of pooled flies showed that the position of the *Df(2L)bhe* breakpoint is variable—as is typical of chromosome ends not capped by a telomere (Biessmann and Mason 1988). The ends fell in the 2L:18,000–19,000 interval within *l(2)gl*. This deletion of the distalmost two genes lies next to a tandem duplication (2L:24,509–25,359) within the adjacent *Ir21a* gene (called *Ir21a*^*bhe*^). We predicted that *CyO* in this stock carries a recessive lethal or sterile mutation in the region immediately proximal to *Ir21a*, so that single crossovers distal to 22D would result in lethal or sterile progeny and stock breakdown would be avoided (Figure 5B).

In crosses combining *CyO* from the *Df(2L)bhe*, *Ir21a*^*bhe*^ stock with a set of molecularly defined deletions encompassing 94% of the genes from the *2L* telomere to the 22D breakpoint (Table S13), we found no recessive lethal or male-sterile mutations, but we identified a recessive female-sterile mutation (*fs(2)21Ba^1^*) in the 15-gene *CG11374*–*ovm* interval immediately proximal to *Ir21a* (Figure S2). In follow-up crosses, we narrowed the position of the mutation to the seven-gene region between *net* and *CG3164*. Sterile females had underdeveloped ovaries with only the occasional mature-looking egg chamber. The rare eggs they laid usually collapsed. We suspect the mutation arose spontaneously in the *Df(2L)bhe*, *Ir21a*^*bhe*^ stock, because the *CyO* chromosomes in 14 related stocks established in the Nüsslein-Volhard lab at roughly the same time lack the mutation (Table S13).

Any recombinant chromosomes arising from single crossovers in the region between *fs(2)21Ba^1^* and the 22D breakpoint would be eliminated from the *Df(2L)bhe*, *Ir21a*^*bhe*^/*CyO*, *fs(2)21Ba^1^* stock. This observation suggests that distal deleterious mutations on balancers are the most likely mechanistic explanation for the unexpected stability of stocks that combine poor balancers of distal regions, such as *CyO* and *TM3*, with chromosomes carrying extremely distal mutations. Indeed, geneticists may have unwittingly selected for balancers with distal mutations through routine stockkeeping practices. In all likelihood, the suppression of meiotic recombination that occurs near telomeres (Hawley 1980; Comeron *et al.* 2012) and tight linkage work together to prevent crossovers between two distal *trans*-heterozygous mutations, such as *Ir21a*^*bhe*^ and *fs(2)21Ba^1^*, from being a significant risk for generating recombinant chromosomes lacking mutations that would lead to stock breakdown. Figure S3 explores the specific case of *Ir21a*^*bhe*^–*fs(2)21Ba^1^* recombination in detail.

## Discussion

This is the fourth in a series of papers (Miller *et al.* 2016b, 2016a, 2018a) aimed at understanding the genomic structure of balancer chromosomes, demonstrating genetic variation among balancers, and illuminating the effectiveness of balancers in maintaining mutation-bearing chromosomes in stable stocks. These studies have shown that balancers, as arguably the most widely and frequently used tools in Drosophila genetics, are interesting both for their utility and the ways by which they inform us about biological processes.

Balancers owe their usefulness to the simple fact that the likelihood of a meiotic recombination event is reduced in the vicinity of aberration breakpoints. Multiple inversions both reduce crossing over between balancers and unrearranged chromosomes and prevent the recovery of most recombinant chromosomes that are formed—though rare, two-strand double crossovers within large regions not interrupted by breakpoints do result in the exchange of sequences (Miller *et al.* 2016b, 2016a, 2018a). Here, we provided molecular detail regarding two important component inversions that were challenging to characterize molecularly, *In(3R)C* and *In(2L)Cy*. Amusingly, *In(3R)C* was the last inversion on a major balancer to have its breakpoints defined at single-nucleotide resolution, but it was the first inversion discovered. Muller (1918) identified *In(3R)C* as a crossover suppressor (*C* = *Crossover suppressor*) on a chromosome isolated from a wild population. He showed that a recessive lethal mutation (*l(3)a^1^*) that arose spontaneously within *In(3R)C* allowed the recessive lethal mutation *Ser*^*Bd-1*^ on a homologous chromosome to be maintained in the first balanced stock. Schwartz (2008) provides an excellent discussion of the historic importance of this discovery and the critical role a clear understanding of inversions and balanced lethal systems played in disproving nonmendelian theories of inheritance and evolution. Coincidentally, the other inversion whose breakpoints were sequenced in this study, *In(2L)Cy*, is one of the two inversions on the second balancing chromosome described (Ward 1923). The characterization of this balancer was an important achievement and is an underappreciated contribution to the history of genetics by an early twentieth-century female scientist.

The precise breakpoints of *In(3R)C* and *In(2L)Cy* could not be determined by short-read sequencing in previous studies (Miller *et al.* 2016a, 2018a) because they are associated with repetitive genomic sequences. The chromatin conformation capture data of Ghavi-Helm *et al.* (2019) was key to narrowing the positions of the inversion ends so that long-read sequence runs spanning the breakpoints could be identified. Inversions isolated from wild Drosophila populations have arisen by two mechanisms: they were either induced by double-strand breaks followed by nonhomologous end-joining, as happened with *In(3R)C*, or by ectopic recombination between repetitive sequences, as happened with *In(2L)Cy* (Ranz *et al.* 2007; Delprat *et al.* 2009; Reis *et al.* 2018; Orengo *et al.* 2019). Curiously, we know of no inversions from wild populations generated by hybrid element insertion, the predominant mode by which inversions have been induced in experiments with *P* elements (Gray *et al.* 1996) and a mechanism by which one might expect inversions to arise after mobilization of *P* element-related *foldback* elements, such as those involved in generating *In(2L)Cy*.

Although the genomic locations of most breakpoints on third chromosome balancers had been determined previously (Miller *et al.* 2016a), we did not know the phenotypic consequences of these breaks, so we examined each breakpoint in complementation tests with deletions. Impressively, most of the breakpoints had no serious effects on viability. This means that the inversions were either homozygous viable and fertile when they were chosen as progenitor chromosomes or they were induced on top of preexisting inversions and kept for their minor effects. Only the *In(3R)C* breakpoint in *FucTA* resulted in severely reduced female fecundity in our tests. In fact, the most recently generated third chromosome balancer, *TM6C*, was homozygous viable and fertile when it was isolated, even though most *TM6C* chromosomes now carry secondary lethal mutations. We have not ascertained how investigators screened for the newly induced inversions on *TM3*, *TM6*, *TM6B* and *TM6C* without relying on overt phenotypes. We suspect they screened for changes in pairing-sensitive interallelic interactions.

We confirmed that breakpoints in he *p53* and *FucTA* genes result in the loss-of-function phenotypes one might expect from disrupting these genes. The *p53* gene is a well-studied tumor suppressor (Zhou 2019; Levine 2020; Tang *et al.* 2020) and *FucTA* affects nervous system development (Yamamoto-Hino *et al.* 2010). While the phenotypes we assayed—failures in apoptosis and protein fucosylation—are more subtle than lethality or abnormal morphological phenotypes, it is easy to see how even heterozygosity for these balancer-borne mutations might affect the interpretation of many experiments.

Likewise, we demonstrated that numerous missense, nonsense and splice-acceptor mutations are shared by balancers and that many others are unique to specific balancers—as one might expect for chromosomes sharing progenitors that have diverged over time. Indeed, balancers, because they undergo limited recombination, provide an excellent “fossil record” of sequence divergence by descent. The *Ank2*^*TM3*^ mutation we identified serves as a marker for a specific lineage of *TM3* chromosomes, and other mutations undoubtedly mark different lineages. While we demonstrated that shared and unique mutations exist on balancers, we did not compare stocks established at a known time with the same balancer. Such an experiment would be interesting with respect to the rates at which mutations accumulate and balancers diverge under routine stock-rearing conditions.

In addition to documenting the accumulation of SNPs and indels, we saw evidence from characterizing *In(3R)C* that balancer chromosomes can be polymorphic for terminal deletions. Cryptic terminal deletions are common in stocks (Mason *et al.* 2004; Roegiers *et al.* 2009), so it is not surprising that some balancers are missing tip sequences. It is easy to imagine terminal deletions preventing the maintenance of distal mutations in stocks or enhancing the phenotypic effects of other mutations, so it is important to consider the potential impact of a terminal deletion when interpreting experimental results.

Finally, our studies provide evidence that distal deleterious mutations can improve the effectiveness of balancers in maintaining the integrity of distal regions on homologous chromosomes. While balancers with distalmost breakpoints positioned far from the telomere, such as *CyO* and *TM3*, usually allow the recovery of recombinant chromosomes from crossovers distal to the breakpoint, our observations indicate that balancers that carry distal deleterious mutations do not. While this mechanism provides a simple, straightforward and perhaps obvious explanation of the unexpected stability of stocks carrying distal mutations, we are not aware of another explicit demonstration.

This report adds detail to our understanding of the most frequently used balancers in Drosophila. We trust our observations improve the usefulness and increase the appreciation of these remarkable chromosomes.
